# Mechanical Properties of Compact Bone Defined by the Stress-Strain Curve Measured Using Uniaxial Tensile Test: A Concise Review and Practical Guide

**DOI:** 10.3390/ma14154224

**Published:** 2021-07-28

**Authors:** Che-Yu Lin, Jiunn-Horng Kang

**Affiliations:** 1 Institute of Applied Mechanics, College of Engineering, National Taiwan University, No. 1, Sec. 4, Roosevelt Road, Taipei 10617, Taiwan; 2 Department of Physical Medicine and Rehabilitation, Taipei Medical University Hospital, 252 Wuxing Str., Taipei 11031, Taiwan; 3 Department of Physical Medicine and Rehabilitation, School of Medicine, College of Medicine, Taipei Medical University, 250 Wuxing Str., Taipei 11031, Taiwan; 4 Research Center of Artificial Intelligence in Medicine, Taipei Medical University, 250 Wuxing Str., Taipei 11031, Taiwan

**Keywords:** bone tissue engineering, hydrogel, construct, orthopedics, orthopaedics, biomechanics

## Abstract

Mechanical properties are crucial parameters for scaffold design for bone tissue engineering; therefore, it is important to understand the definitions of the mechanical properties of bones and relevant analysis methods, such that tissue engineers can use this information to properly design the mechanical properties of scaffolds for bone tissue engineering. The main purpose of this article is to provide a review and practical guide to understand and analyze the mechanical properties of compact bone that can be defined and extracted from the stress–strain curve measured using uniaxial tensile test until failure. The typical stress–strain curve of compact bone measured using uniaxial tensile test until failure is a bilinear, monotonically increasing curve. The associated mechanical properties can be obtained by analyzing this bilinear stress–strain curve. In this article, a computer programming code for analyzing the bilinear stress–strain curve of compact bone for quantifying the associated mechanical properties is provided, such that the readers can use this computer code to perform the analysis directly. In addition to being applied to compact bone, the information provided by this article can also be applied to quantify the mechanical properties of any material having a bilinear stress–strain curve, such as a whole bone, some metals and biomaterials. The information provided by this article can be applied by tissue engineers, such that they can have a reference to properly design the mechanical properties of scaffolds for bone tissue engineering. The information can also be applied by researchers in biomechanics and orthopedics to compare the mechanical properties of bones in different physiological or pathological conditions.

## 1. Introduction

Bone is a specialized organ that provides several important functions for the human body, including supporting the entire body and internal soft tissues, supporting and protecting internal organs, assisting in movement with skeletal muscles, regulating mineral homeostasis (especially for calcium and phosphorus), producing blood cells, and storing triglycerides for energy reserve [1]. Since bone has multiple functions and is composed of several different connective tissues [1], it would be more reasonable to define bone as an organ rather than a tissue, although bone is often described as a tissue. In order to carry out these important functions [2], bone is designed as a complex and dynamic living organ that remodels continuously throughout an individual’s lifetime [1,3,4]. It means that, during our lifetimes, bones continuously undergo a process involving the resorption of old or damaged bones by osteoclasts (bone resorption) and the following formation of new bones by osteoblasts (bone formation) [2,5,6]. Bone remodeling is crucial to adjust bone architecture and mechanical properties to meet mechanical demands, to repair damaged bones, and to prevent increased bone mass due to impaired removal of old bones [7,8]. Bone remodeling is regulated by both biochemical and mechanical factors, in which mechanical loading (stress and strain) plays an important role [9,10]. Specifically speaking, mechanical loading increases signals that recruit osteoblasts and inhibit osteoclasts while decreases signals that recruit osteoclasts and inhibit osteoblasts [6]. Balanced bone remodeling is crucial for maintaining the health and functions of bones, and for the repair and replacement of damaged bones [11,12]. Imbalanced bone remodeling caused by improper coordination between osteoclasts and osteoblasts can result in abnormal bone mass and quality, as well as bone diseases such as osteoporosis and osteopetrosis [13,14].

Bone has a high regeneration potential because of its remarkable remodeling ability [15]. In normal conditions, microdamages of bones often can be successfully regenerated (self-repaired), and the form and function of bones can be restored once the regeneration is completed [16]. However, fractures or large defects of bones typically could not be properly regenerated and need to be treated by clinical intervention, such as casting or surgical reconstruction [16]. Traditional clinical practices for reconstructing bone defects include the transplantation of autografts (transplanting tissue from a donor site to the recipient site on the same patient) and allografts (transplanting tissue from an individual to the patient) [16,17,18,19]. These two techniques have been used in clinical practice for years and have been successful in saving lives, but they have some major problems. Harvesting autografts is expensive, and may result in problems associated with trauma such as pain, infection, hematoma, and necrosis on the donor site [16,17,18,20]. The condition and availability of the donor tissue may also limit the feasibility of using autografts [18]. On the other hand, although using allografts avoids the need for an additional surgery to harvest the donor tissue from the patient, it may cause risks of rejection by the patient’s immune system and transmission of diseases from the donor to the patient. In addition, low availability of allografts is also a major concern [21]. In addition to the use of autografts and allografts, metal-based and ceramic-based implants are therapeutic approaches often used to repair bone defects [19]. However, their clinical application values could be limited because of their relatively low biochemical and biomechanical compatibility with native bones. Therefore, although metal and ceramic implants could restore the structure and shape of the bone, they may not effectively restore the function and provide long-term therapeutic effectiveness.

Tissue engineering provides an alternative approach to regenerate damaged bones [22,23]. Instead of reconstructing damaged tissues using conventional surgical interventions such as autografts, allografts, or implants, tissue engineering aims to replace damaged tissues by engineered tissues produced using porous scaffolds seeded with cells [18]. Cell-seeded scaffolds are designed to mimic the extracellular matrix of natural tissues [24,25], providing the appropriate environment for the growth of cells and then the formation of engineered tissues. Cell-seeded scaffolds can be cultured in vitro to synthesize tissues that will then be transplanted into the damaged site; alternatively, cell-seeded scaffolds can be implanted directly into the damaged site, and the formation of engineered tissues will be induced in vivo using the body’s own cells and growth factors [18]. The use of engineered tissues to regenerate damaged tissues can avoid the shortcomings of conventional surgical interventions described in the previous paragraph.

Hydrogels are ideal materials for scaffolds since they have similar properties to the extracellular matrix of natural tissues [24,25,26,27,28]. Hydrogels, typically made of natural or synthetic polymers as well as large amounts of water [29,30], are gel-like materials that consist of three-dimensional networks of cross-linked polymer chains in which water is the medium [31,32]. Since the physical, chemical, compositional, structural and functional properties of hydrogels may be manipulated and custom-designed, they are ideal candidates for mimicking the extracellular matrix and for producing the environment for the growth of cells [26]. The design of the hydrogel-based scaffolds must consider a number of key factors, including biocompatibility, biodegradability, chemical, compositional, structural and mechanical properties [18]. These factors are all important for all tissue types, and must be carefully designed and tuned during the design and fabrication processes. For bones, a proper design of the mechanical properties of engineered bone tissues is particularly important. Once a scaffold is implanted into the bone damaged site, the scaffold must stimulate and support continuous cell growth as well as subsequent tissue remodeling and maturation [19]. In addition, the scaffold must provide sufficient initial mechanical functions and vascularization for the damaged bone, and then degrade at a rate that is compatible with the regeneration of new tissues [19,33,34,35]. Finally, the regenerated bone from the scaffold should restore the mechanical functions of the natural native bone to withstand physiological mechanical loadings. The fulfillment of the above-mentioned requirements needs proper mechanical properties of scaffolds [16]. It has been reported that the mechanical properties of scaffolds have significant effects on cell behaviors, including cell attachment, proliferation, and differentiation [36]. Therefore, since mechanical properties are crucial parameters for scaffolds for bone tissue engineering, it is important to understand the definitions of the mechanical properties of bones and relevant analysis methods, such that tissue engineers can use this information to properly design the mechanical properties of scaffolds for bone tissue engineering [37].

The main purpose of this article is to provide a review and practical guide to understand and analyze the mechanical properties of compact bone defined by the stress–strain curve measured using uniaxial tensile test until failure. In this article, firstly, we briefly review the composition and structure of bone tissue, and review two types of bone, namely, compact and spongy bones. Then, we review the mechanical properties of compact bone that can be defined and extracted from the stress–strain curve measured using uniaxial tensile test until failure. The typical stress–strain curve of compact bone measured using uniaxial tensile test until failure is a bilinear, monotonically increasing curve. The associated mechanical properties can be obtained by analyzing this bilinear stress–strain curve. In this article, a computer programming code for analyzing the bilinear stress–strain curve of compact bone for quantifying the associated mechanical properties is provided, such that the readers can use this computer code to perform the analysis directly. In addition to being applied to compact bone, the information provided by this article can also be applied to quantify the mechanical properties of any material having a bilinear stress–strain curve, such as a whole bone, some metals and biomaterials. The information provided by this article can be applied by tissue engineers, such that they can have a reference to properly design the mechanical properties of scaffolds for bone tissue engineering. The information can also be applied by researchers in biomechanics and orthopedics to compare the mechanical properties of bones in different physiological or pathological conditions.

## 2. Composition and Structure of Bone Tissue

In this section, we briefly review the composition and structure of a bone tissue. The content in this section is mainly referred to Refs. [1,38,39].

The mechanical properties of bone tissue, the primary tissue that makes up bone, are primarily determined by the composition and structure of bone tissue. It is important to remind that whole bone is actually an organ consisting of several different connective tissues including bone tissue. Please note that, in the context below, the term “bone” means “bone tissue” but not “bone organ”.

Compositionally speaking, bone is a composite material made up of organic and inorganic components. Organic components make up around 40% of the bone’s dry weight, and the primary organic component of bone is collagen fiber (mainly type I collagen). Inorganic components (in the form of mineral salts) make up around 60% of the bone’s dry weight, and the primary inorganic component is hydroxyapatite (a calcium phosphate–based mineral). Like other connective tissues, bone contains an abundant extracellular matrix. The organic and inorganic components construct the extracellular matrix of bone in a way that the framework of the extracellular matrix is formed by collagen fibers (organic components) while hydroxyapatite materials (inorganic components) are deposited on the framework for crystallizing and hardening the framework. The extracellular matrix of bone also contains around 25% water and several types of cells including osteoprogenitor cells, osteocytes, osteoblasts and osteoclasts.

**Figure 1:**
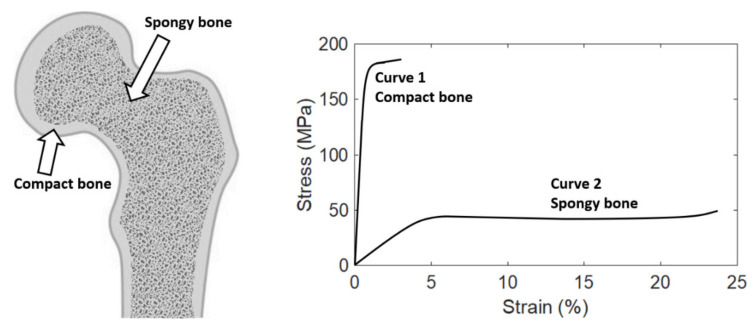
**Left**: Bone tissue can be categorized as two types, compact bone and spongy bone. **Right**: The typical stress–strain curves of compact and spongy bones. The last point of the stress–strain curve is the failure point. This figure is adapted from [39].

Structurally speaking, bone has many small spaces (pores). Based on the size and density of the small space, bone can be categorized as two types, namely, compact bone (also called cortical bone) and spongy bone (also called trabecular bone), as shown in Figure 1. The relative quantity of each type differs among bones, but on average, compact bone and spongy bone constitute around 80% and 20% of the skeleton, respectively. These two types of bone have identical composition, but are different in structure macroscopically and microscopically. Compact bone forms the outer shell (or called cortex, and that is the reason why compact bone is also called “cortical” bone) of whole bone. The spaces within compact bone are much smaller; therefore, compact bone is much denser with a porosity of 5–10% and apparent density of 1.5–1.8 g/cm^3^ (that is the reason why it is called “compact” bone). Spongy bone is located at the end or on the inside of whole bone, and is surrounded by the outer compact bone. Spongy bone is composed of thin columns called trabeculae (that is the reason why spongy bone is also called “trabecular” bone), and is loose and less dense with a porosity of 50–90% and apparent density of 0.5–1.0 g/cm^3^. The porosity is one of the factors that strongly affect the mechanical properties of bone. Therefore, compact and spongy bones have significantly different mechanical properties because of their significant difference in the porosity. Compact bone can withstand much higher stress (up to about 150 MPa) but lower strain (up to about 3%) before failure, while spongy bone can withstand lower stress (up to about 50 MPa) but much higher strain (up to about 50%) before failure [40,41,42,43,44].

Normal whole bone is stiff and strong, but it is flexible and ductile, not brittle. This bidirectional mechanical behavior is contributed by the composition and structure of whole bone described above. Compositionally speaking, the inorganic components of bone make bone stiff and strong, while the organic components offer bone flexibility, ductility and toughness. Structurally speaking, compact bone is much stiffer and stronger than spongy bone, while spongy bone is more flexible and ductile. Therefore, the overall mechanical behavior of whole bone is the combination of these two diverse behaviors, making bone stiff and strong but at the same time flexible and ductile. This specialized mechanical behavior makes whole bone a versatile tissue having multiple mechanical functions, such as support, protection, and shock absorption.

## 3. Mechanical Properties of Compact Bone

In this section, we review the mechanical properties of compact bone that can be defined and extracted from the stress–strain curve measured using uniaxial tensile test until failure.

The stress–strain curve of a material represents the relationship between the stress and strain of that material under loading, and can be obtained by material testing system, either using uniaxial tensile or compression tests. The mechanical properties of a material can be obtained by analyzing the stress–strain curve of that material. For more information about the concepts of stress and strain as well as the standard method for obtaining the stress–strain curve of a material by a material testing system, please refer to [45].

The typical stress–strain curves of compact and spongy bones measured using uniaxial tensile test until failure are shown in Figure 1. It can be observed that the stress–strain curves of these two types of bone are quite different. Compact bone is much stiffer but more brittle than spongy bone. It means that compact bone can withstand much higher stress but less strain than spongy bone before failure. In addition, spongy bone can withstand much more energy (quantified by the area under the stress–strain curve) before failure, thanks to its porous structure.

**Figure 2:**
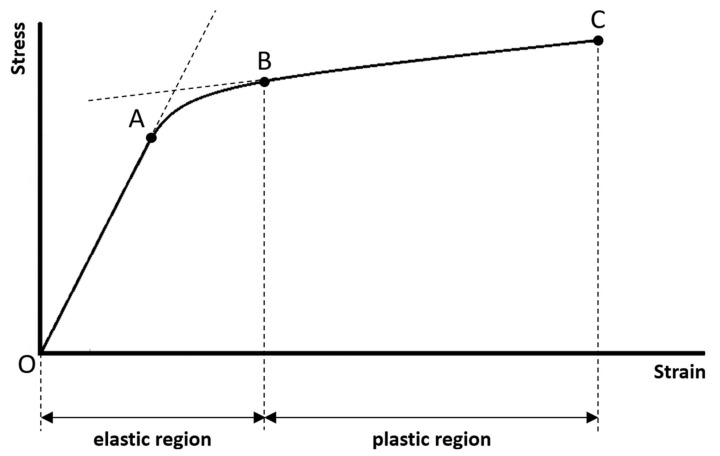
The typical stress–strain curve of compact bone measured using uniaxial tensile test until failure. Two regions (elastic and plastic regions) and four points (points O, A, B, and C) that have significant mechanical meanings are marked.

The typical stress–strain curve of compact bone measured using uniaxial tensile test until failure is highlighted in Figure 2. It is important to note that this stress–strain curve is a bilinear, monotonically increasing curve. There are two linear curves in this bilinear curve, and that is the reason why a curve with this pattern is called a bilinear curve. The first linear curve (from points O to A, i.e., the linear curve within the elastic region) has a significantly greater slope than the second linear curve (from points B to C, i.e., the linear curve within the plastic region). Between the two linear curves is a short nonlinear curve. There are some regions and points associated with this stress–strain curve that have significant mechanical meanings, marked in Figure 2. These regions and points are closely related to the mechanical properties defined by the stress–strain curve. Below, we sequentially review a series of mechanical properties (listed in Table 1) of compact bone that can be defined and extracted from the stress–strain curve.

(1) Range of the elastic region:

The elastic region is between points O (this is the origin of the stress–strain curve with zero stress and strain that indicates the instant of the beginning of loading) and B (the mechanical meaning of point B will be explained below). If the sample is loaded within the elastic region, the stress and strain will be completely recovered and back to zero once the applied loading is removed. There will be no permanent stress and strain, and the sample will be intact without any damages as well as compositional and structural changes, if the sample is loaded within the elastic region. In mechanics of material, the term “elasticity” is defined as the ability of a material to resume its original size and shape once the applied loading is removed. The greater the range of the elastic region, the greater the ability of the sample to reserve its elasticity.

(2) Range of the plastic region:

The plastic region is between points B and C (the mechanical meanings of points B and C will be explained below). If the sample is loaded beyond the elastic region and into the plastic region, there will be permanent strain (or called plastic strain) even though the applied loading is removed. The permanent strain is due to the permanent compositional and structural changes of the sample. It means that the sample will undergo damages as well as permanent compositional and structural changes, if the sample is loaded beyond the elastic region and into the plastic region. In mechanics of material, the term “plasticity” not only can imply damage and permanent strain, but also can imply ductility before failure. The greater the range of the plastic region, the greater the ductility of the sample. It means that the sample can undergo greater strain before failure; therefore, the sample may have a much lower chance to fail suddenly.

(3) Proportional limit:

The stress at point A is called the proportional limit. Although the proportional limit is not a point but actually means the stress at point A, one is accustomed to call it a point for the convenience of communication. Point A marks the end of the first linear curve. Between points O and A, the stress–strain curve is linear and is a straight line. It means that the stress and strain are linearly proportional on this curve. Beyond point A, the proportionality between the stress and strain no longer exists, and this is the reason why the stress at point A is called the proportional limit. In literature, some authors may assume that the proportional limit is equal to the elastic limit (the elastic limit will be explained below). It is reasonable to make such an assumption, since the proportional limit is often very close to the elastic limit for a material, although they are two different concepts. However, in this article, we suggest to assume that the proportional limit is not equal to the elastic limit.

(4) Elastic limit:

The stress at point B is called the elastic limit. Although the elastic limit is not a point but actually means the stress at point B, one is accustomed to call it a point for the convenience of communication. Point B marks the beginning of the second linear curve, also marks the end of the elastic region and the beginning of the plastic region. It means that point B is the boundary just between the elastic and plastic regions. This is the reason why the stress at point B is called the elastic limit. Beyond point B, the sample is loaded into the plastic region and undergoes damages as well as permanent compositional and structural changes. The stress and strain corresponding to point B are the largest stress and strain that can be applied to the sample without causing any permanent strain. If the sample is loaded beyond point B, it will not resume its original size and shape even though the applied loading is removed. It is important to note that most of the authors in literature prefer to use the term “yield point” to call point B, but not use the term “elastic limit”. However, we suggest that using the term “elastic limit” to indicate point B is more accurate, since point B marks the end of the elastic region. Besides, at least for compact bone, no significant yielding phenomenon (strain increases significantly while there is no observed increase in stress) can be observed on this stress–strain curve. It is also important to note that the elastic limit is seldom constantly defined in literature, and has been determined by different methods by different authors [46]. For example, the elastic limit is often defined as the same as proportional limit (point A). The offset method is another method sometimes used to determine the elastic limit, in which a line parallel to the first linear curve of the stress–strain curve is constructed, and the elastic limit is defined as the intersection of this line and the stress–strain curve. Please refer to [45] for more information about the offset method. In this article, we suggest to define the elastic limit as the beginning of the second linear curve (Point B).

(5) Failure strength:

Point C is called the failure point that marks the occurrence of the failure. The stress at point C is called the failure strength. In mechanics of materials, failure is defined as the state at which the sample completely breaks into more than one piece. The failure strength is the maximum stress that the sample can withstand before failure. The maximum stress in a stress–strain curve is called the ultimate strength; therefore, failure strength is equal to the ultimate strength in this case.

(6) Brittleness coefficient:

The brittleness coefficient is defined as the ratio of the strain at point B (i.e., the strain corresponding to the elastic limit) to the strain at point C (i.e., the strain where the failure occurs):

(1) $$\mathrm{brittleness}\;\mathrm{coefficient}=\frac{\mathrm{strain}\;\mathrm{corresponding}\;\mathrm{to}\;\mathrm{the}\;\mathrm{elastic}\;\mathrm{limit}}{\mathrm{strain}\;\mathrm{where}\;\mathrm{the}\;\mathrm{failure}\;\mathrm{occurs}}$$

The brittleness coefficient is used to quantify how brittle the sample is, and it is a number between 0 and 1. The more the brittleness coefficient is close to 1, the more brittle the sample is. In order to understand what that means, it is important to remind what a brittle or a ductile material is. A material can be classified as either brittle or ductile in terms of how great the range of the plastic region is, compared to the range of the elastic region. A brittle material fails at a relatively low strain without undergoing a significant permanent strain, typically once the elastic limit is just reached. A ductile material undergoes a large permanent strain before failure. Therefore, the brittleness coefficient can be used to indicate the ratio between the range of the elastic region and the range of the plastic region. If the range of the elastic region is much greater than the range of the plastic region, the brittleness coefficient is greater, and the sample is more brittle.

**Figure 3:**
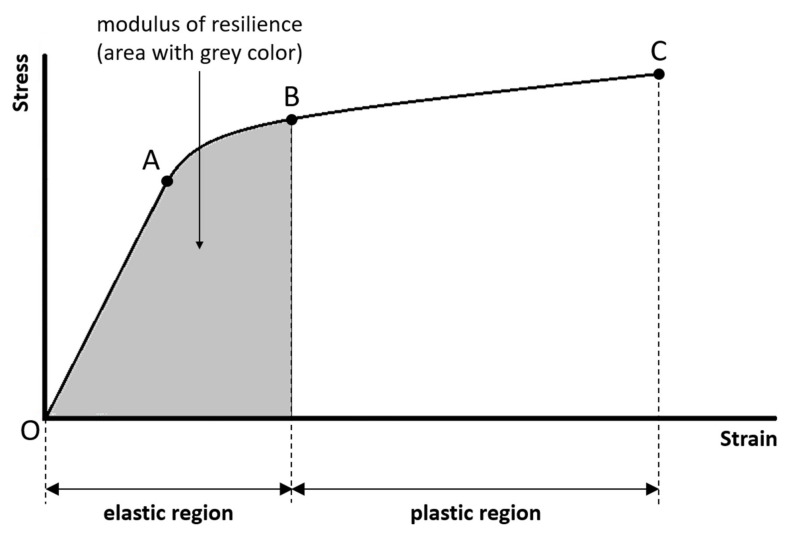
The modulus of resilience is the amount of energy per unit volume necessary to cause damages as well as permanent compositional and structural changes to the sample, and is quantified by the area under the stress–strain curve in the elastic region.

(7) Modulus of resilience:

The modulus of resilience is the amount of energy per unit volume necessary to cause damages as well as permanent compositional and structural changes to the sample. The modulus of resilience is quantified by the area under the stress–strain curve in the elastic region (Figure 3). The greater the modulus of resilience, the greater the ability of the sample to absorb energy without permanent strain. The ability of a material to absorb energy without permanent strain is called resilience.

**Figure 4:**
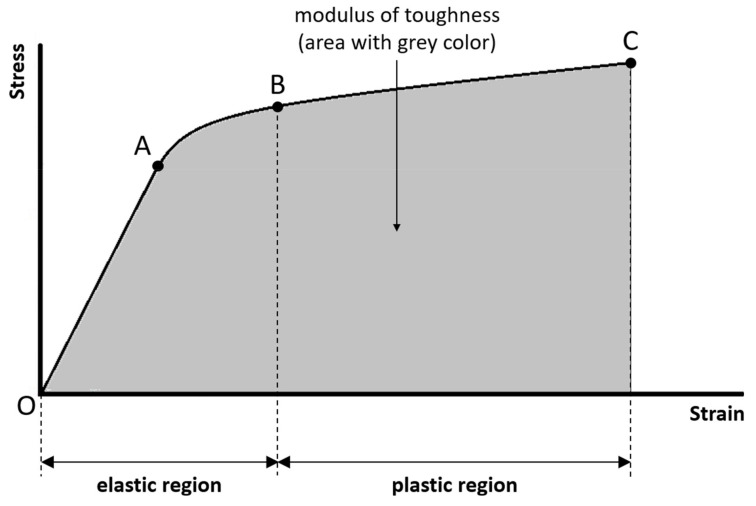
The modulus of toughness is the amount of energy per unit volume necessary to completely break the sample, and is quantified by the area under the entire stress–strain curve.

(8) Modulus of toughness:

The modulus of toughness is the amount of energy per unit volume necessary to completely break the sample. The modulus of toughness is quantified by the area under the entire stress–strain curve (Figure 4). The greater the modulus of toughness, the greater the ability of the sample to absorb energy without failure. The ability of a material to absorb energy without failure is called toughness.

(9) Modulus of elasticity:

The modulus of elasticity is the slope of the first linear curve, and it is a parameter used to quantify how stiff the sample is within the elastic region. The greater the modulus of elasticity, the stiffer the sample and the greater the resistance to loading within the elastic region.

(10) Tangent modulus:

The tangent modulus is the slope of the second linear curve, and it is a parameter used to quantify how stiff the sample is within the plastic region. The greater the tangent modulus, the stiffer the sample and the greater the resistance to loading within the plastic region. The term “hardening” is used to indicate the phenomenon that the stress increases with the increasing strain within the plastic region. Therefore, the tangent modulus is also called the strain-hardening modulus, used to quantify the degree of hardening. The greater the tangent modulus, the greater the degree of hardening. The tangent modulus must be smaller than the modulus of elasticity, and could be zero. If the tangent modulus is zero (i.e., the second linear curve is horizontal), the plastic property of the material is said to be perfectly plastic.

(11) Strain hardening parameter:

In addition to using the tangent modulus to quantify the degree of hardening, there is another parameter called the strain hardening parameter that can be used to quantify the degree of hardening. The strain hardening parameter is defined as:

(2) $$\mathrm{strain}\;\mathrm{hardening}\;\mathrm{parameter}=\frac{E\cdot E_{T}}{E-E_{T}}$$

where E is the modulus of elasticity, and $E_{T}$ is the tangent modulus. The greater the strain hardening parameter, the greater the degree of hardening. If $E_{T}$ is equal to E, the strain hardening parameter approaches infinity; however, this case cannot happen in reality, since the tangent modulus must be smaller than the modulus of elasticity. If $E_{T}$ is equal to zero, the strain hardening parameter is equal to zero, and this corresponds to the case that the plastic property of the material is perfectly plastic.

**Figure 5:**
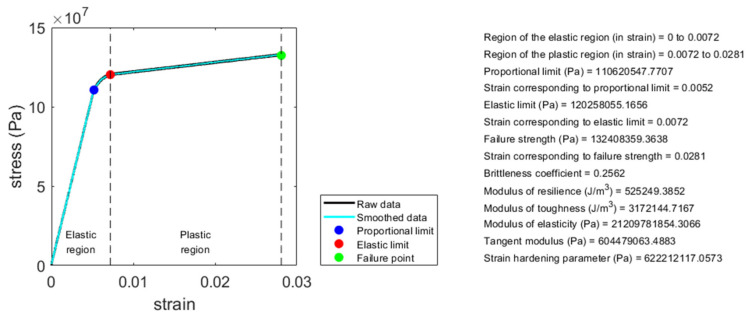
Illustration of an example of the analysis result using the MATLAB computer programming code provided along with this article. By analyzing the bilinear stress–strain curve of a material using this computer code, the associated mechanical properties can be quantified.

The eleven parameters introduced above can be used to quantify the mechanical properties of any material having a bilinear stress–strain curve, including those of compact bone. In this article, a MATLAB (Mathworks, Natick, MA, USA) computer programming code for analyzing the bilinear stress–strain curve for quantifying these eleven mechanical properties is provided. The readers can use this computer code to analyze these eleven mechanical properties of any material having a bilinear stress–strain curve, including compact bone. Please see the Appendix A for the link to download the computer code and relevant information. Figure 5 shows an example of the analysis result using this computer code. The data shown in Figure 5, adapted from FIGURE 1-19 in [47], is a stress–strain curve of a compact bone sample measured using uniaxial tensile test until failure. This data is provided along with the computer code, serving as an example data for the readers to use the computer code.

## 4. Discussion

In this article, we provide a review and practical guide to understand and analyze the mechanical properties of compact bone defined by the stress–strain curve measured using uniaxial tensile test until failure. In addition, a computer programming code for analyzing the bilinear stress–strain curve of compact bone for quantifying the associated mechanical properties is provided, such that the readers can use this computer code to perform the analysis directly. In addition to being applied to compact bone, the information provided by this article can also be applied to quantify the mechanical properties of any material having a bilinear stress–strain curve, such as a whole bone, some metals and biomaterials.

The information provided by this article can be applied by tissue engineers, such that they can have a reference to properly design the mechanical properties of scaffolds for bone tissue engineering. As mentioned in the Introduction section, a proper design of the mechanical properties of scaffolds is important, since the mechanical properties determine several crucial factors such as the mechanical functions of scaffolds and the effects on cell behaviors and tissue remodeling. Therefore, the implanted scaffold with proper mechanical properties can have sufficient functions for tissue remodeling through the entire remodeling process [18,33]. In addition, a proper design of the mechanical properties of scaffolds can also help avoid detrimental conditions related to mechanical mismatch, such as stress shielding, implantation-related osteopenia, and fracture [17]. Mechanical mismatch, or mismatch in mechanical properties, is a problem that often occurs in the use of traditional metal and ceramic implants, but thanks to the rapidly advancing field of bone tissue engineering, engineered bone tissues with proper mechanical properties can provide a promising alternative solution for overcoming that problem. Therefore, a thorough understanding and proper design of the mechanical properties of bone is one of the keys to the success of scaffolds for bone tissue engineering, although there are other important factors (such as the porous architecture of scaffolds that may affect the capacity for cell infiltration and vascularization) needed to be considered as well [18]. However, it is important to note that bone has a complex hierarchical structure, in which the structures and mechanical properties change continuously at different length scales [44,47,48]. In addition, the mechanical properties of bone are anisotropic (i.e., orientation-dependent) [44,47]. Therefore, for designing an optimal scaffold, one might need to understand the mechanical properties of a whole bone, compact and spongy bones, single collagen fibril, single osteon and lamellae, the organic and inorganic components, and so on, under different orientations of loading. Studies at each of these length scales can provide valuable insight into the mechanical properties and functions of bone.

**Figure 6:**
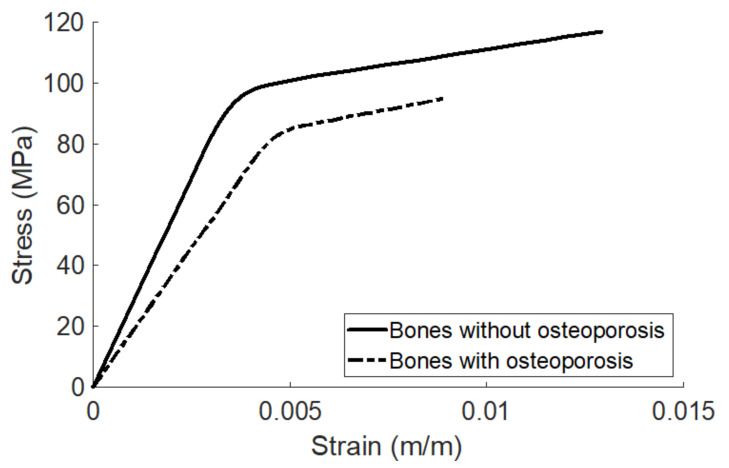
The stress–strain curves of bones with and without osteoporosis. This figure is adapted from Figure 4 in [49].

The information provided by this article can be applied to whole bone as well, since the patterns of the stress–strain curve of compact bone and whole bone are similar, both exhibiting bilinear behavior. Therefore, in addition to be applied in the field of bone tissue engineering, the information provided by this article can also be applied by researchers in biomechanics and orthopedics to compare the mechanical properties of whole bones in different conditions. The stress–strain curves of whole bones in different physiological or pathological conditions may all exhibit as bilinear, but the patterns of the curves and the associated mechanical properties may be different. Since there is a one-to-one functional relationship between the stress–strain curve and the associated mechanical properties, the pattern of the stress–strain curve specifically determines the associated mechanical properties. It means that two identical stress–strain curves yield two identical sets of mechanical properties, and on the other hand, two different stress–strain curves yield two different sets of mechanical properties. For example, it has been shown that the stress–strain curves of bones with and without osteoporosis measured using uniaxial tensile test are both bilinear but have significantly different patterns [49], as shown in Figure 6 (adapted from the Figure 4 in [49]). Table 2 shows the mechanical property parameters of the two stress–strain curves in Figure 6 analyzed using the computer code provided along with this article. The trend of analysis is consistent with that of [49]. It can be observed that a bone without osteoporosis is stiffer (the modulus of elasticity is higher), stronger (failure strength is higher), more ductile (the brittleness coefficient is lower), more resilient (the modulus of resilience is higher), and tougher (the modulus of toughness is higher), compared to a bone with osteoporosis. It means that a bone with osteoporosis can be broken more easily compared to a bone without osteoporosis, since the amounts of stress, strain, and energy needed to cause failure for a bone with osteoporosis are significantly lower. The elastic limit of a bone without osteoporosis is higher, meaning that a bone without osteoporosis can sustain a higher stress before loaded into the plastic region, compared to a bone with osteoporosis. However, it is interesting to note that a bone with osteoporosis has a higher tangent modulus and strain hardening parameter, meaning that a bone with osteoporosis is somewhat stiffer in the plastic region, compared to a bone without osteoporosis. In addition, the range of the elastic region of a bone with osteoporosis is wider than a bone without osteoporosis, meaning that a bone with osteoporosis can sustain a higher strain before loaded into the plastic region.

**Figure 7:**
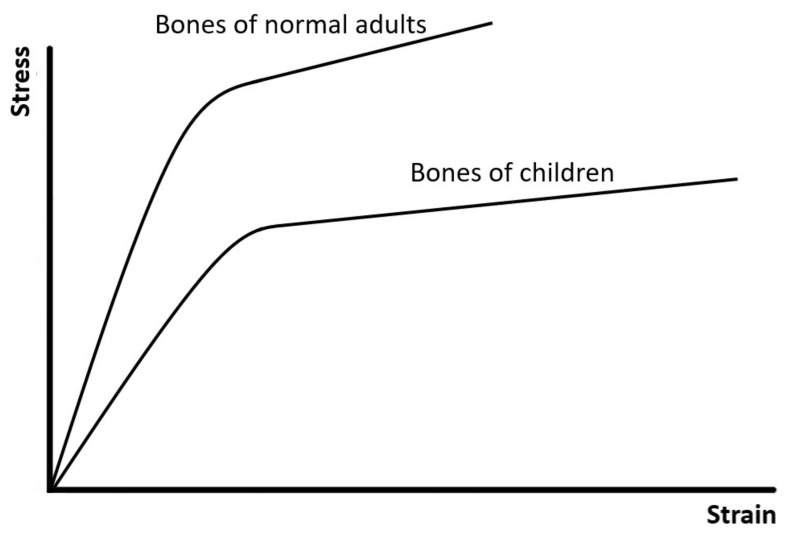
The stress–strain curves of bones of normal adults and children. This figure is adapted from [46].

Figure 7 shows another example, illustrating that bones of normal adults and children have different patterns of bilinear stress–strain curves and different mechanical properties [46]. Compared to bones of normal adults, bones of children are softer but more ductile and tougher since they have not been completely mineralized. Therefore, the bone of a child sometimes may undergo a large deformation but would not completely fracture.

In addition to be applied to compare the mechanical properties of whole bones in different physiological or pathological conditions, the information provided by this article can also be applied to evaluate the effectiveness of treatment methods on pathological bones. Different treatment methods may result in different bilinear stress–strain curves and associated mechanical properties of bones. Therefore, by analyzing the bilinear stress–strain curves resulted from different treatment methods, their treatment effectivenesses can be quantified in terms of the mechanical properties.

In addition to be applied to quantify the mechanical properties of any material having a bilinear stress–strain curve, the information provided by this article can also be applied to quantify the nine mechanical properties (No. 1 to 9 in Table 1, except for the tangent modulus and strain hardening parameter) for a non-bilinear stress–strain curve having a linear curve in the elastic region but the curve in the plastic region is not linear. For example, based on some research findings [39], the pattern of the stress–strain curve of compact bone measured using uniaxial compression test, or that of spongy bone measured using uniaxial tensile test, may appear like curve No. 2 shown in Figure 1. Even though it is not a typical bilinear curve because the curve in the plastic region is not linear, it has a linear curve in the elastic region. Therefore, the information provided by this article can be applied to quantify some of their mechanical properties, including the ranges of the elastic and plastic regions, proportional and elastic limits, failure strength, brittleness coefficient, modulus of resilience, modulus of resilience, modulus of toughness, and modulus of elasticity. However, since its curve in the plastic region is not linear, the tangent modulus and strain hardening parameter are not defined and cannot be quantified. It is important to note that the pattern of the stress–strain curve of a material depends on several factors, including the type of material, location of the sample taken from the material, size and physiological condition of the sample, type of material testing system, orientation of the loading, details and settings of the experimental method, and environmental conditions during testing (such as temperature and humidity). Therefore, the stress–strain curve of a specific type of material might not always exhibit a specific pattern. For example, the pattern of the stress–strain curve of compact bone measured using uniaxial compression test, or that of spongy bone measured using uniaxial tensile test, might not always appear like that of curve No. 2 shown in Figure 1. It has been reported that the stress–strain curve of spongy bone is less consistent and predictable, owing to its highly variable and less organized porous structure [41,44,50,51,52,53,54,55,56,57]. Regardless of the type and condition of materials, as long as the stress–strain curve is bilinear, or at least the curve in the elastic region is linear, the information provided by this article can be applied to quantify all or some of the mechanical properties reviewed in this article.

The mechanical properties of compact bone reviewed in this article are those measured using uniaxial tensile test. To understand the mechanical properties of a bone tissue in response to tensile loadings is important clinically, since tensile loadings can result in some common types of fractures. For example, tendons and ligaments constantly cause tensile loadings at their attachment sites on the bones. In some abnormal conditions, an avulsion fracture may occur at the attachment site of a tendon or ligament on the bone due to that tensile loading. Two examples are fractures of the base of the fifth metatarsal adjacent to the attachment of the peroneus brevis tendon, and fractures of the calcaneus adjacent to the attachment of the Achilles tendon [38]. However, it is worth mentioning that it is equally important to understand the mechanical properties of a bone tissue in response to compressive, tensile, and shear loadings, since these three loading modes can lead to different types of fractures [38]. In addition, bones constantly sustain these three loading modes during activities of daily living or due to accidental scenarios such as a trauma [53,54,58,59,60,61,62]. The mechanical properties of a bone tissue are anisotropic, meaning that the mechanical properties of a bone tissue are different along different orientations and a bone tissue will respond differently if different orientations of loadings are applied [44,47]. The stress–strain curve of compact bone measured using uniaxial compression or shear test might not be a perfect bilinear curve, since, typically, the part of the curve measured using these two loading modes in the plastic region is not linear, even though that in the elastic region might be linear [39]. However, it might be approximated as a bilinear curve in some circumstances, if the nonlinear curve in the plastic region could be reasonably approximated as a linear curve (based on the subjective intuition of the decision maker or an objective method). In such circumstances, all of the mechanical properties (including the tangent modulus and strain hardening parameter) of compact bone measured using uniaxial compression test can still be quantified using the information provided by this article, if a certain amount of error is allowed.

In conclusion, the main purpose of this article is to provide a review and practical guide to understand and analyze the mechanical properties of compact bone defined by the stress–strain curve measured using uniaxial tensile test until failure. The typical stress–strain curve of compact bone measured using uniaxial tensile test until failure is a bilinear, monotonically increasing curve. The associated mechanical properties can be obtained by analyzing this bilinear stress–strain curve. In this article, a computer programming code for analyzing the bilinear stress–strain curve of compact bone for quantifying the associated mechanical properties is provided, such that the readers can use this computer code to perform the analysis directly. In addition to being applied to compact bone, the information provided by this article can also be applied to quantify the mechanical properties of any material having a bilinear stress–strain curve, such as a whole bone, some metals and biomaterials. The information provided by this article can be applied by tissue engineers, such that they can have a reference to properly design the mechanical properties of scaffolds for bone tissue engineering. The information can also be applied by researchers in biomechanics and orthopedics to compare the mechanical properties of bones in different physiological or pathological conditions.

## Figures and Tables

**Table 1:** Mechanical properties of compact bone that can be defined and extracted from the stress–strain curve.

	Name of the Mechanical Property Parameter	Unit
1	Range of the elastic region (in strain)	Dimensionless
2	Range of the plastic region (in strain)	Dimensionless
3	Proportional limit (in stress)	Pa
4	Elastic limit (in stress)	Pa
5	Failure strength (in stress)	Pa
6	Brittleness coefficient	Dimensionless
7	Modulus of resilience	J/m^3^
8	Modulus of toughness	J/m^3^
9	Modulus of elasticity	Pa
10	Tangent modulus	Pa
11	Strain hardening parameter	Pa

**Table 2:** Mechanical properties of bones with and without osteoporosis.

	Name of the Mechanical Property Parameter	Bones with Osteoporosis	Bones without Osteoporosis
1	Range of the elastic region (in strain) (m/m)	0–0.0063	0–0.0043
2	Range of the plastic region (in strain) (m/m)	0.0063–0.0089	0.0043–0.0129
3	Proportional limit (in stress) (MPa)	77.0934	80.3718
4	Elastic limit (in stress) (MPa)	88.3528	98.6828
5	Failure strength (in stress) (MPa)	94.9280	116.9657
6	Brittleness coefficient (Dimensionless)	0.7079	0.3333
7	Modulus of resilience (MJ/m^3^)	0.3394	0.2450
8	Modulus of toughness (MJ/m^3^)	0.5778	1.1751
9	Modulus of elasticity (MPa)	18283.2314	27544.2425
10	Tangent modulus (MPa)	2490.2230	2118.0671
11	Strain hardening parameter (MPa)	2882.8784	2294.5076

## Data Availability

The data presented in this study are available on reasonable request from the corresponding author.

## Supplementary Materials

The relevant MATLAB code are available online at https://drive.google.com/drive/folders/19Nia3j1hogZl_UoMLbaXGYiybFajkWWS?usp=sharing.

## Appendix A

In this article, a MATLAB computer programming code is provided for analyzing the bilinear stress–strain curve of compact bone (or any material having a bilinear stress–strain curve) for quantifying the associated mechanical properties. This computer code, a note for explaining how to use this code, and an example data can be downloaded from the Supplementary Materials along with this article, or from the following link: https://drive.google.com/drive/folders/19Nia3j1hogZl_UoMLbaXGYiybFajkWWS?usp=sharing (accessed on 28 July 2021).
